# Kaempferol Alleviates Steatosis and Inflammation During Early Non-Alcoholic Steatohepatitis Associated With Liver X Receptor α-Lysophosphatidylcholine Acyltransferase 3 Signaling Pathway

**DOI:** 10.3389/fphar.2021.690736

**Published:** 2021-06-28

**Authors:** Hongjiao Xiang, Mingmei Shao, Yifei Lu, Junmin Wang, Tao Wu, Guang Ji

**Affiliations:** ^1^ Institute of Interdisciplinary Integrative Medicine Research, Shanghai University of Traditional Chinese Medicine, Shanghai, China; ^2^ Institute of Digestive Disease, Longhua Hospital, Shanghai University of Traditional Chinese Medicine, Shanghai, China

**Keywords:** kaempferol, NASH, LXR, LPCAT3, ERS

## Abstract

**Background:** Kaempferol (KP) has a variety of biological effects such as anti-inflammatory, anti-oxidant, anti-aging and cardiovascular protection. Whether KP has a therapeutic effect on non-alcoholic steatohepatitis (NASH), and the detailed mechanism is currently unclear. This study aims to explore the mechanism of KP in the treatment of NASH through *in vivo* and *in vitro* experiments.

**Methods:** 1) *In vivo* experiment: In the C57BL/6 NASH mice model induced by high fat diet (HFD), KP was administered by gavage at a dose of 20 mg/kg/day. 2) *In vitro* experiment: Palmitic acid/Oleic acid (PA/OA, 0.375/0.75 mM) was used to intervene HepG2 and AML12 cells to establish a steatosis cell model. Three concentrations of KP, low (20 μmol/L), medium (40 μmol/L) and high (60 μmol/L) were used *in vitro*. The mRNA and protein expression of related molecules involved in LXRα-LPCAT3-ERS pathway were detected using RT-qPCR and Western blot.

**Results:** In the NASH mouse model, KP can significantly reduce the expression of LXRα, LPCAT3 and ERS-related factors PERK, eIF2α, ATF6, ATF4, XBP1, CHOP, IRE1α and GRP78. In the PA/OA-induced cell model, KP could decrease the content of triglyceride and lipid droplets, and also decrease the expression of LXR α, LPCAT3 and ERS related factors PERK, eIF2α, ATF6, ATF4, XBP1, CHOP, IRE1α and GRP78.

**Conclusion:** KP may decrease the expression level of LXRα and LPCAT3, thus improve ERS and reduce hepatic steatosis and inflammation.

## Introduction

Non-alcoholic fatty liver disease (NAFLD) is a clinical pathological syndrome, which includes simple steatosis, steatohepatitis (NASH), liver fibrosis and cirrhosis and their clinical consequences (Nobili et al., 2019). NASH is usually associated with more severer liver cirrhosis and liver cancer (Friedman et al., 2018). NAFLD is one of the leading cause of liver disease in the world, which is closely linked to the increased prevalence of obesity and metabolic diseases. NAFLD affects more than 20% of the population of the Europe and United States. NAFLD prevalence varies widely from the Asia-pacific region, ranging from about 5 to 32% (Younossi et al., 2018). Approximately one third of NAFLD patients will progress to NASH (Takahashi and Fukusato, 2014).

Phospholipids (PLs) is an important part of biological membranes and the precursors of many signal molecules (Vance, 2015). Liver X receptors (LXRs) are regulators of membrane phospholipid composition, important regulators for regulating cholesterol and fatty acid homeostasis, and effective inflammation inhibitors (Rong et al., 2013). Lysophosphatidylcholine acyltransferase 3 (LPCAT3) is a key enzyme that encodes the remodeling pathway to PLs, and is a direct target gene of mouse and human LXR (Demeure et al., 2011; Wang et al., 2016). LPCAT3 modulates the activation of inflammatory kinases by changing membrane components, and regulates inflammation by affecting the availability of substrates produced by inflammatory mediators (Singh and Liu, 2017b). Rong et al. stated that LXR activation increased the expression of LPCAT3 and reduced the saturation of the ER membrane. Acute knockout of LPCAT3 in the liver could aggravate endoplasmic reticulum stress (ERS). At the same time, the inflammation of the liver would increase because the activity of LPCAT3 was inhibited. It can be concluded that the LXR-LPCAT3 pathway is important to regulating ERS, and is involved in the progress of NASH.

Apoptosis is an active cell death process controlled by gene, which is mainly divided into endogenous pathway (mitochondrial pathway), exogenous pathway (death receptor pathway) and ERS induced apoptosis (Hu et al., 2018). The link between ERS and liver disease has been found in a growing number of studies, including NAFLD, alcoholic fatty liver, viral hepatitis, NASH, liver cancer, etc., (Brenner et al., 2013; Li F. et al., 2017). Endoplasmic reticulum (ER) is the main site for protein synthesis, lipid production and calcium ion storage in eukaryotic cells. When cells are stimulated externally, the ER produces a large number of unfolded or misfolded proteins, which leads to the unfolded protein response (UPR) (Chaudhari et al., 2014; Demirtas et al., 2016). In the early stage, UPR can relieve the pressure on the ER and promote the restoration of normal function of ER. However, sustained ERS induces apoptosis through UPR (Ozcan and Tabas, 2012). Cell signal transduction pathways induced by UPR include three pathways: protein kinase R-like ER kinase/eukaryotic translation initiation factor 2 (PERK/eIF-2α), activating transcription factor 6 (ATF6) pathway and inositol-requiring enzyme 1/X-box-binding protein 1 (IRE1/XBP1) (Wang and Kaufman, 2016). ERS activates sterol regulatory element binding protein (SREBP) through the ATF6, PERK and IRE1 signaling pathways to promote lipid accumulation in the liver (Zhang et al., 2012). It has been proved that ERS can activate the apoptotic pathway and induce apoptosis by activating downstream signal molecules (Iurlaro and Muñoz-Pinedo, 2016). Dasgupta et al. (2020) demonstrated that the destruction of IRE1A or inhibition of Ire1a in liver cells reduces the release of inflammatory extracellular vesicles (EVs), and reduces liver damaged and inflammation in the mice were fed a diet high in fat, fructose, and cholesterol. Bax inhibitor-1 (BI-1) is a negative regulator of the ER stress sensor IRE1α. Lebeaupin et al. (2018) treated BI-1^−/−^ mice on a high-fat diet (HFD) with tunicamycin and developed liver failure of a short period of time. After IRE1α inhibitors were used to treat BI-1^−/−^ mice, liver inflammation was significantly improved. Hernández-Alvarez et al. (2019) found that the lack of Mfn2 would promote ERS, leading to the progression of NASH to liver cancer. Therefore, regulating ERS can be considered as an important approach and new strategy for prevention and treatment of steatosis.

Kaempferol (KP) is a dietary bioflavonoid, which is widely present in various plants. It has a variety of biological effects such as anti-inflammatory, anti-oxidant, anti-aging and cardiovascular protection (Li and Pu, 2011; Lee B. et al., 2015; Suchal et al., 2017; Kouhestani et al., 2018). Lee B. et al. (2015) found that KP may bind to PPARα during adipocyte differentiation to stimulate fatty acid oxidation signals in adipocytes, thereby reducing cellular triglyceride (TG) accumulation. Lee Y.-J. et al. (2015) used zebrafish to verify the inhibitory effect of KP on lipid accumulation. KP inhibited lipid accumulation in zebrafish by inhibiting fat-related genes (Lee B. et al., 2015). Wang et al. (2019) found that KP can improve liver failure induced by lipopolysaccharide-galactosamine by reducing ERS. Other studies have shown that the regulation of KP on ERS was bidirectional, which can promote the apoptosis of tumor cells and inhibit the apoptosis of non-tumor cells (Ashrafizadeh et al., 2020). Our previous review summarized the application of KP in treating diseases and the underlying mechanisms that are currently being studied (Ren et al., 2019). In our previous study, it has been demonstrated that KP not only reduced serum alanine aminotransferase (ALT), low density lipoprotein (LDL), TG and total cholesterol (TC) content, but also reduced the accumulation of lipids in the liver (Lu et al., 2020). However, there are insufficient studies on the detailed mechanism of KP on improving NASH.

In this study, we revealed that the expression of LXR-LPCAT3-ERS pathway-related factors was remarkably increased to the liver of mice fed a HFD, and the similar results were observed in palmitic acid/oleic acid (PA/OA)-induced steatotic cells. After intervention with KP, either *in vitro* or *in vivo*, KP significantly reduced the expression of LXR-LPCAT3 pathway, inhibited the expression of molecules involved in ERS, and then improved HFD-related liver damage. Finally, we conclude that KP is a promising drug for the treatment of NASH.

## Materials and Methods

### Animals and Treatment

Six-week-old male C57BL/6J mice were purchased from Shanghai SLAC Experimental Animal Co., Ltd. China. The experimental animals are kept in the specific pathogen free environment of the Experimental Animal Center of Shanghai University of Traditional Chinese Medicine. The temperature is about 20 ± 4°C, and the humidity is about 15–16%. C57BL/6J mice were randomly divided into normal control group (Control, NC group, N = 7), HFD (Calorie composition: fat 60%, protein 20%, carbohydrate 20%, D12492, Research Diets, United States) fed group (HFD model, HFD group, N = 8) and HFD fed with KP (HFD + KP, HFD + KP group, N = 8). The mice in group NC were fed normal diet, and the mice in group HFD and HFD + KP were fed HFD for 24 weeks. At the beginning of the 21st week, KP (4 mg/ml) was gavaged in the HFD + KP group at a dose of 0.5 ml/100 g on the basis of HFD feeding. At the same time, mice in NC and HFD groups received equal volume of normal saline (Supplementary Figure S1). The serum and liver tissue of mice was collected. All the experimental procedures were approved by the Institutional Animal Care and Use Committee at the Shanghai University of Traditional Chinese Medicine (SYXKhu 2014-0008).

### Cell Lines

HepG2 and AML12 cells were obtained from the Cell bank of typical culture preservation Committee of Chinese Academy of Sciences. The cells were cultured in high glucose DMEM (containing 10% fetal bovine serum, 100 U/ml penicillin, 100 μg/ml streptomycin and 1‰ Amphotericin B) and was maintained at 37°C, 95% air humidity and 5% CO_2_.

### Establishment of Steatosis Cell Line

PA (P0500, Sigma, United States) was dissolved in methanol, and OA (0550, Sigma, United States) was dissolved in PBS. PA:OA was then added to the culture solution at a ratio of 1:2. Final concentrations of PA/OA were as below: ①0 mM, ②0.25/0.5 mM, ③0.375/0.75 mM, ④0.5/1 mM, ⑤0.625/1.25 mM and ⑥0.75/1.5 mM. HepG2 and AML12 cells were cultured for 24 h to observe the morphological changes. Cells were detected with cell counting kit-8 (CCK8), TG kit and Oil-Red O staining.

### Real-Time Quantitative PCR

Total RNA was extracted from mouse livers, HepG2 and AML12 cells using Trizol reagents (R0016, Beyotime Biotechnology, Shanghai, China). cDNA were transcribed according to the instructions in the reverse transcription kit (A5001, Promega, United States). The cDNA was using real-time quantitative polymerase chain reaction (RT-qPCR) performed with the SYBR Green PCR Master Mix (11201E03, YEASEN, Shanghai, China) and gene-specific primers. The results were normalized with β-actin as control RT-qPCR was performed using a CFX-96 Real Time System (Bio-Rad, United States). Polymerase chains reaction primers are listed in Supplementary Table S1.

### Western Blot

Liver and cell sample were lysed with radio immunoprecipitation assay (RIPA) buffer (WB0102, Weiao, Shanghai, China) containing phenylmethylsulfonyl fluoride (PMSF) (WB0122, Weiao, Shanghai, China). The proteins were extracted after centrifugation, and subsequently a bicinchoninic acid (BCA) (B48110, YEASEN, Shanghai, China) protein quantitation kit was used to determine the concentrations. 30 μg of protein was subjected to 10% sodium dodecyl sulfate-polyacrylamide (SDS-PAGE) gel electrophoresis, then transferred to a polyvinylidene fluoride membrane, and then incubated with the corresponding primary antibody overnight at 4°C. Antibodies and dilution ratio were as below: 1:1,000 for rabbit anti-Actin (R1207, HUABIO, Hangzhou, China), 1:1,000 for rabbit anti-LXRα (ab176323, Abcam, Cambridge, MA), 1:500 for rabbit anti-LPCAT3 (HAPL0516, HUABIO, Hangzhou, China), 1:1,000 for rabbit anti-phospho-PERK (3,179, Cell Signaling Technology, MA), 1:1,000 for rabbit anti-PERK (20582-1-AP, Proteintech Group, MA), 1:1,000 for rabbit anti-phospho-eIF2α (3398S, Cell Signaling Technology, MA), 1:1,000 for rabbit anti-eIF2α (3398S, Cell Signaling Technology, MA), 1:500 for rabbit anti-ATF4 (10835-1-AP, Proteintech Group, MA), 1:200 for rabbit anti-CHOP (15204-1-AP, Proteintech Group, MA), 1:500 for rabbit anti-ATF6 (ab203119, Abcam, Cambridge, MA), 1:1,000 for rabbit anti-GRP78 (3177S, Cell Signaling Technology, MA), 1:1,000 for rabbit anti-phospho-IRE1α (AF7150, Affbiotech, China), 1:1,000 for rabbit anti-IRE1α (3294S, Cell Signaling Technology, MA), 1:1,000 for rabbit anti-XBP1 (ab220783, Abcam, Cambridge, MA), 1:200 for rabbit anti-PPARα (ab215270, Abcam, Cambridge, MA), 1:200 for rabbit anti-ABCA1 (ab18180, Abcam, Cambridge, MA). The membrane was then incubated with a secondary antibody (1:5,000, Peroxidase-conjugated affinity goat anti-mice IgG, HA1001, HUABIO, China). ImageJsoftware (National Institutes of Health, Bethesda, MD) was used to scan and analyze the western blot band to obtain protein quantitative data.

### Cell Proliferation Assay

The cells were planted in 96-well plates with a density of 1 × 10^^4^ cells per well. After the cells were cultured for 24 h, 10 μl of CCK8 solution was added to each well of the 96-well plate and mixed. After in a 37°C incubator for 3 h, the optical density was measured at 450 nm wavelength with a microplate reader (Infinite M200 PRO, TECAN, Switzerland).

### Triglyceride Detection

The cells were planted in 96-well plates with a density of 1 × 10^^4^ cells per well. After 24 h of incubation, the cell suspension was extracted, and TG was detected according to the instructions in the intracellular TG kit (A110-1, Jiancheng, Nanjing, China).

### Oil Red O Staining

The cells were cultured in a 12-well plate with a cell density of 2 × 10^^5^ cells per well. After the cells were cultured for 24 h, the culture medium in the 12-well plate was discarded. The cells were washed twice with PBS and fixed with 4% paraformaldehyde for 30 min. The fixatives were removed in the 12-well plate and cells were washed twice with PBS. Oil Red O staining solution (G1262, Solarbio, Beijing, China) was added to the 12-well plate and incubate for 30 min. The cells were washed with 60% isopropanol and stained with hematoxylin for 30 s. Finally, the cells were rinsed three times with distilled water.

### Statistical Analysis

All experimental data were analyzed using GraphPad Prism version 8.0, and all data are presented as the means ± SEM. One-way analysis of variance assessment with Tukey’s test and Bonferroni’s post hoc test was used to statistically analyze differences among groups. *p <* 0.05 was considered significant.

## Results

### Kaempferol Significantly Reduced the Expression of Liver X Receptors α and Lysophosphatidylcholine Acyltransferase 3 in High-Fat Diet-Induced Non-Alcoholic Steatohepatitis Mouse Model

**FIGURE 1:**
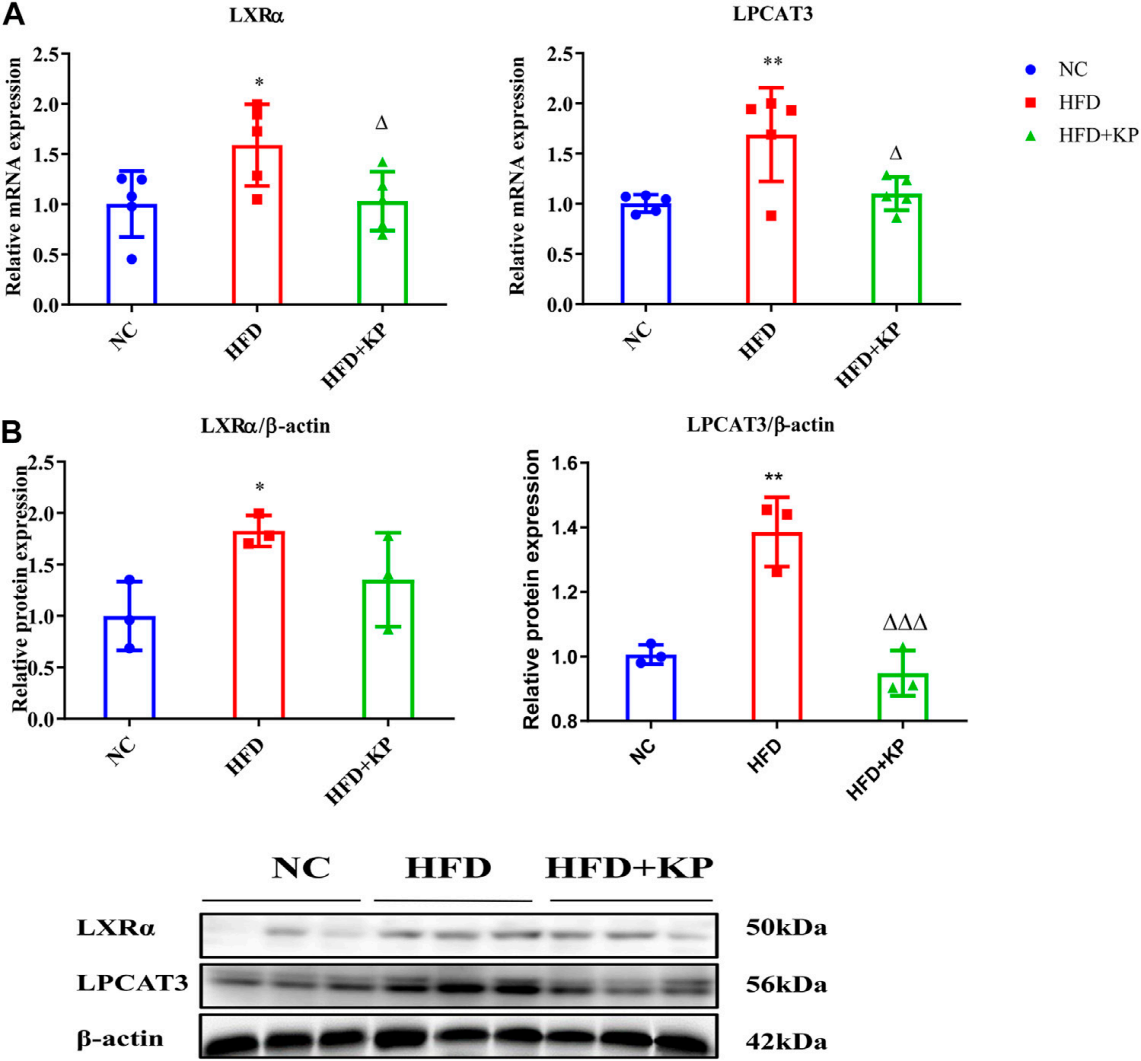
KP regulated the mRNA and protein expression of LXRα and LPCAT3 *in vivo*. **(A)**: mRNA level expression of LXRα and LPCAT3 in liver samples; **(B)**: Protein level expression of LXRα and LPCAT3 in liver samples. NC: Normal control group, HFD: HFD group, HFD + KP: HFD + KP group. Quantification of mRNA and protein level expression was normalized to ß-actin levels. Compared with group NC, *, *p <* 0.05, **, *p <* 0.01; Compared with group HFD, △, *p <* 0.05.

In order to study whether KP played a role in the LXRα-LPCAT3-ERS pathway, the mRNA and protein expression of LXRα and LPCAT3 levels in liver in the NASH mice model were first studied. Compared with group NC, the mRNA expression of LXRα and LPCAT3 in the liver in group HFD were significantly higher (*p <* 0.05 and *p <* 0.01, respectively) (Figure 1A). Compared with the HFD group, the expression of LXRα and LPCAT3 mRNA in the group HFD + KP was drastically decreased (*p <* 0.05 and *p <* 0.05, respectively) (Figure 1A). The similar result was obtained at the protein level. However, though the protein expression of LXRα in group HFD + KP was lower than that in group M, there was no statistical difference (Figure 1B).

### Kaempferol Regulated the Expression of Factors Related to the Endoplasmic Reticulum Stress Signaling Pathway in High-Fat Diet-Induced Non-Alcoholic Steatohepatitis Mouse Model

**FIGURE 2:**
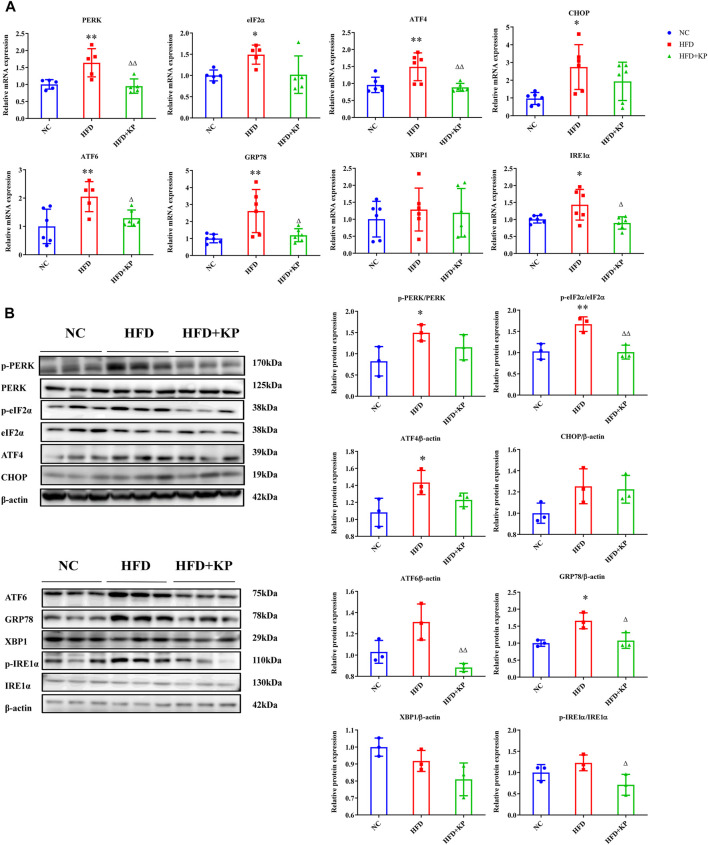
KP regulated the mRNA and protein expression of factors related to ERS *in vivo*. **(A)**: mRNA expression in liver samples; **(B)**: protein expression in liver samples. NC: Normal control group, HFD: HFD group, HFD + KP: HFD + KP group. For **(A,B)**, Quantification of mRNA and protein level expression was normalized to β-actin levels, except protein phosphorylation level expression of PERK, eIF2α, IRE1α expression was normalized to their prototypes. Compared with group NC, *, *p <* 0.05, **, *p <* 0.01; Compared with group HFD, △, *p <* 0.05, △△, *p <* 0.01.

Next, we further analyzed the expression of related molecules in the following step involved in ERS. RT-qPCR results showed that the mRNA levels of PERK, eIF2α, ATF4, CHOP, ATF6, GRP78 and IRE1α in the liver in the HFD group were significantly increased than thoses in NC group. After KP intervention, the mRNA expressions of PERK, ATF4, ATF6, GRP78 and IRE1α were significantly reduced. However, there was no statistically significance of the mRNA levels of eIF2α, CHOP and XBP1 (Figure 2A).

At the protein level, compared with the NC group, the phosphorylation levels of PERK and eIF2α in the liver in the HFD group have been extensively multiplied (*p <* 0.05 and *p <* 0.01, respectively), and the protein expression of ATF4 and GRP78 also elevated notably (*p <* 0.05). However, the protein expression of ATF6, XBP1 and IRE1α in group HFD did not change appreciably compared with group NC. After KP intervention, the phosphorylation levels of eIF2α and IRE1α were significantly reduced, and the protein expression levels of eIF2α, ATF6 and GRP78 were also significantly reduced. The differences between PERK, ATF4, CHOP and XBP1 expression among groups were not statistically significant (Figure 2B). Collectively, these results indicate that KP can protect liver from ERS damage induced by HFD.

### Kaempferol Reduced the mRNA Expression of Inflammatory Factors in the High-Fat Diet-Induced Non-Alcoholic Steatohepatitis Mouse Model

**FIGURE 3:**
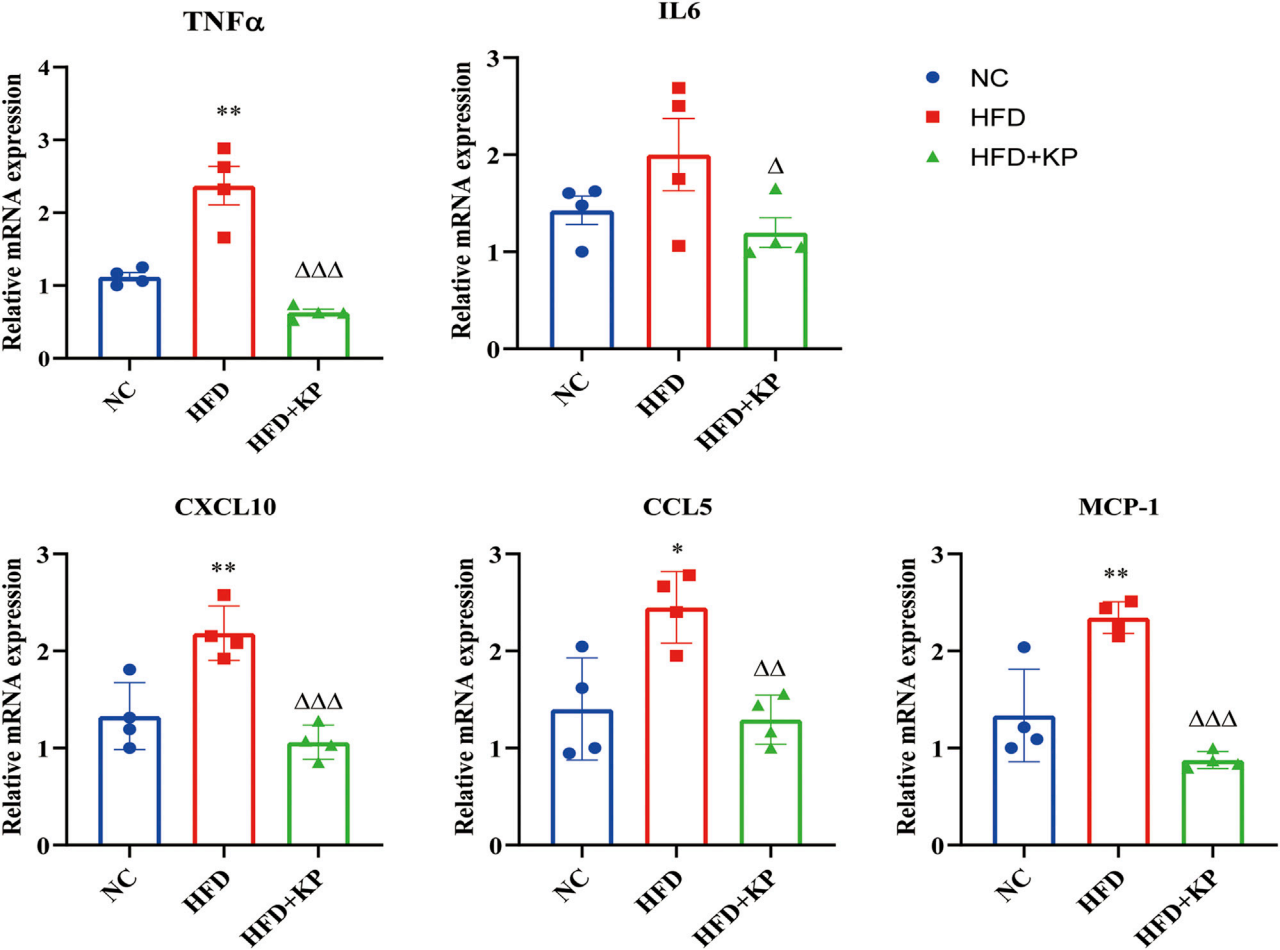
KP reduced the mRNA expression of inflammatory factors *in vivo*. NC: Normal control group, HFD: HFD group, HFD + KP: HFD + KP group. Quantification of mRNA level expression was normalized to β-actin levels. Compared with group NC, *, *p <* 0.05, **, *p <* 0.01; Compared with group HFD, △, *p <* 0.05, △△, *p <* 0.01, △△△, *p <* 0.001.

In order to find out whether KP can improve the state of NASH, qRT-PCR was used to detect the mRNA expression of inflammation-related factors in liver tissues. Compared with the NC group, the expressions of tumor necrosis factor α (TNFα), C-X-C motif chemokine 10 (CXCL10), C-C chemokine ligand 5 (CCL5) and monocyte chemoattractant protein-1 (MCP-1) mRNA in the HFD group increased, and interleukin 6 (IL6) had a rising trend, but there was no statistical difference. After KP treatment, the mRNA expression levels of TNF-α, IL6, CXCL10, CCL5 and MCP-1 in the HFD + KP group were significantly reduced (Figure 3).

### Kaempferol Reduced the Deposition of Lipid Droplets in Cells Induced by Palmitic Acid/Oleic Acid

**FIGURE 4:**
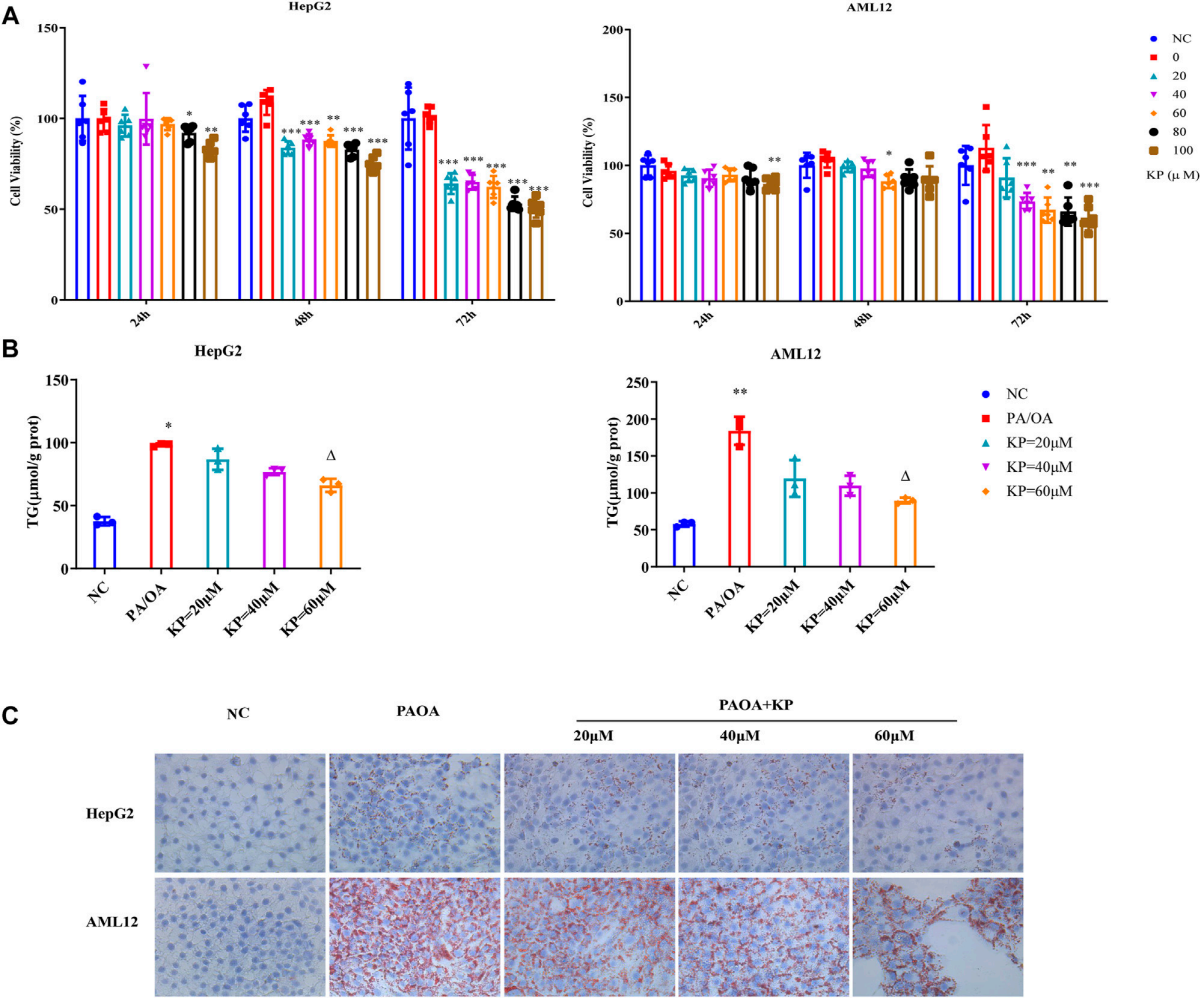
KP reduced the deposition of lipid droplets in cells induced by PA/OA. **(A)**: The survival rate of HepG2 and AML12 cells after the intervention of KP in different concentration and time; **(B)**: The change of TG content after different concentration of KP in HepG2 and AML12 cells interfered with PA/OA (0.375/0.75 mM); **(C)**: The oil red O staining in HepG2 and AML12 cells interfered with PA/OA (0.375/0.75 mM) and/or KP with different concentrations (× 400). NC: Normal control group, PA/OA: PA/OA group, KP = 20 µM: KP low concentration group, KP = 40 µM: KP medium concentration group, KP = 60 µM: KP high concentration group. Compared with group NC, *, *p <* 0.05, **, *p <* 0.01, ***, *p <* 0.001; Compared with group PA/OA, △, *p <* 0.05.

In order to verify the efficacy of KP on NASH, we further established an *in vitro* steatosis model. First of all, the best simulation condition of PA/OA and the best intervention condition of KP in cells were selected. PA/OA was used to establish the model in HepG2 and AML12 cells. The three test results of CCK8 cell viability detection, oil red O staining method and cell TG content were comprehensively analyzed. We found that when the PA/OA concentration was 0.375/0.75 mM, the activity of HepG2 and AML12 cells was not significantly affected. At the same time, a large number of intracellular lipid droplets were formed, and the intracellular TG content was significantly higher than that of the normal control group (Supplementary Figure S2). Therefore, 0.375/0.75 mM PA/OA was selected as the best concentration for modeling. To evaluate the effect of KP on cell viability, KP at concentrations of 0, 20, 40, 60, 80, and 100 μM were added to HepG2 and AML12 cells, and then were cultured for 24, 48, and 72 h, respectively. It was finally found that when the concentration was not higher than 60 μM and within a 24 h time, KP was safe and non-toxic to HepG2 and AML12 cells. Therefore, this concentration range and time were selected as the intervention conditions for subsequent KP intervention experiments (Figure 4A).

Based on the above conditions, it was divided into normal control group (NC), PA/OA group (PA/OA 0.375/0.75 mM), KP low-dose group (PA/OA 0.375/0.75 mM + KP 20 μM), KP medium dose group (PA/OA 0.375/0.75 mM + KP 40 μM), KP high-dose group (PA/OA 0.375/0.75 mM + KP 60 μM) *in vitro*. In different dose groups of KP, PA/OA and KP were added to the medium at the same time and cultured for 24 h. The results confirmed that, in contrast with group NC, TG content in HepG2 cells and AML12 cells in group PA/OA were significantly increased (*p <* 0.05 and *p* < 0.01, respectively). After treatment with KP at three concentrations of 20, 40 and 60 μM, the TG content of HepG2 and AML12 cells decreased, and the concentration decreased notably at 60 μM (*p <* 0.05) (Figure 4B). Oil red O staining showed that the deposition of lipid droplets in HepG2 and AML12 cells in the PA/OA group increased compared with that in the NC group, and KP significantly reduced lipid deposition in both cells (Figure 4C).

### Kaempferol Reduced the Expression of Liver X Receptors α and Lysophosphatidylcholine Acyltransferase 3 in Palmitic Acid/Oleic Acid-Induced Steatosis Model Cells

It has been validated that KP can reduce the expression of LXRα and LPCAT3 in liver tissue, and an *in vitro* model has been used to further verify this result.

**FIGURE 5:**
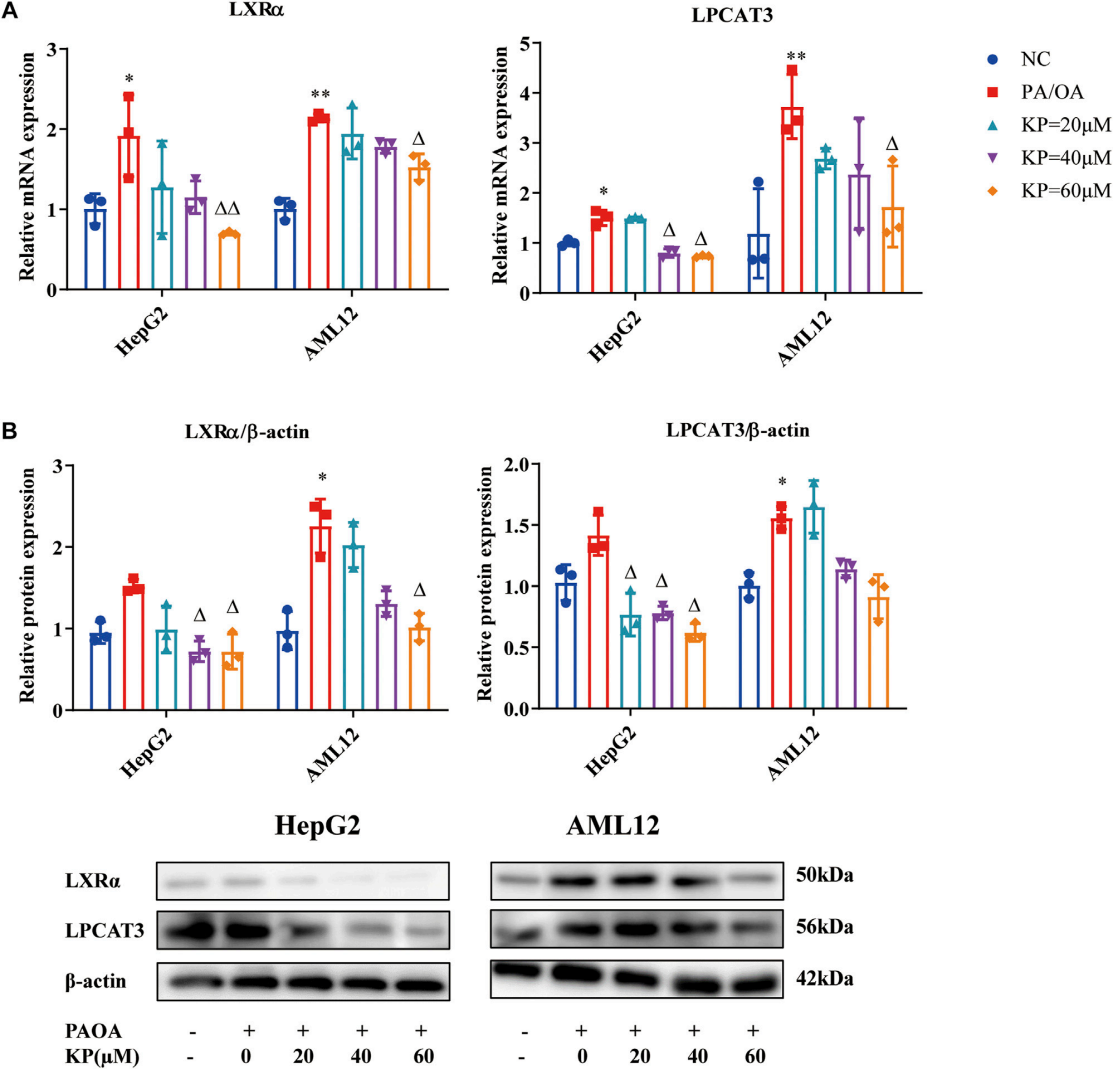
KP regulated the mRNA and protein expression of LXRα and LPCAT3 *in vitro*. **(A)**: mRNA level expression of LXRα and LPCAT3 in HepG2 and AML12 cells; **(B)**: Protein level expression of LXRα and LPCAT3 in HepG2 and AML12 cells. NC: Normal control group, PA/OA: PA/OA group, KP = 20 µM: KP low concentration group, KP = 40 µM: KP medium concentration group, KP = 60 µM: KP high concentration group. Quantification of mRNA and protein level expression was normalized to ß-actin levels. Compared with group NC, *, *p <* 0.05, **, *p <* 0.01; Compared with group PA/OA, △, *p <* 0.05, △△, *p <* 0.01.

The mRNA levels of LXRα and LPCAT3 in the PA/OA group were substantially up-regulated in both HepG2 and AML12 cells induced by PA/OA. After KP intervention, the mRNA transcription of LXRα and LPCAT3 were significantly down-regulated (Figure 5A).

Western blot results showed that the level of LXRα protein increased in the two cells in the PA/OA group, and it was more obvious in AML12 cells (*p* < 0.05). After the intervention of different concentrations of KP, the LXRα protein levels of the two cells were all down-regulated (Figure 4B). Compared with group NC, the protein level of LPCAT3 in each cell of group PA/OA was increased, while it was significantly increased in AML12 cells (*p* < 0.05) (Figure 5B). The protein level of LPCAT3 in the two cells decreased in a concentration-dependent manner when the KP concentration was 40 and 60 μM. It can be seen from the above results that KP can significantly reduce the expression of LXRα and LPCAT3 in the steatosis model.

### Kaempferol Regulated the Expression of Factors Related to the Endoplasmic Reticulum Stress Signaling Pathway in Palmitic Acid/Oleic Acid-Induced Steatosis Model Cells

Subsequently, the mRNA expression and protein expression levels of the ERS-related factors of HepG2 and AML12 cells were detected.

**FIGURE 6:**
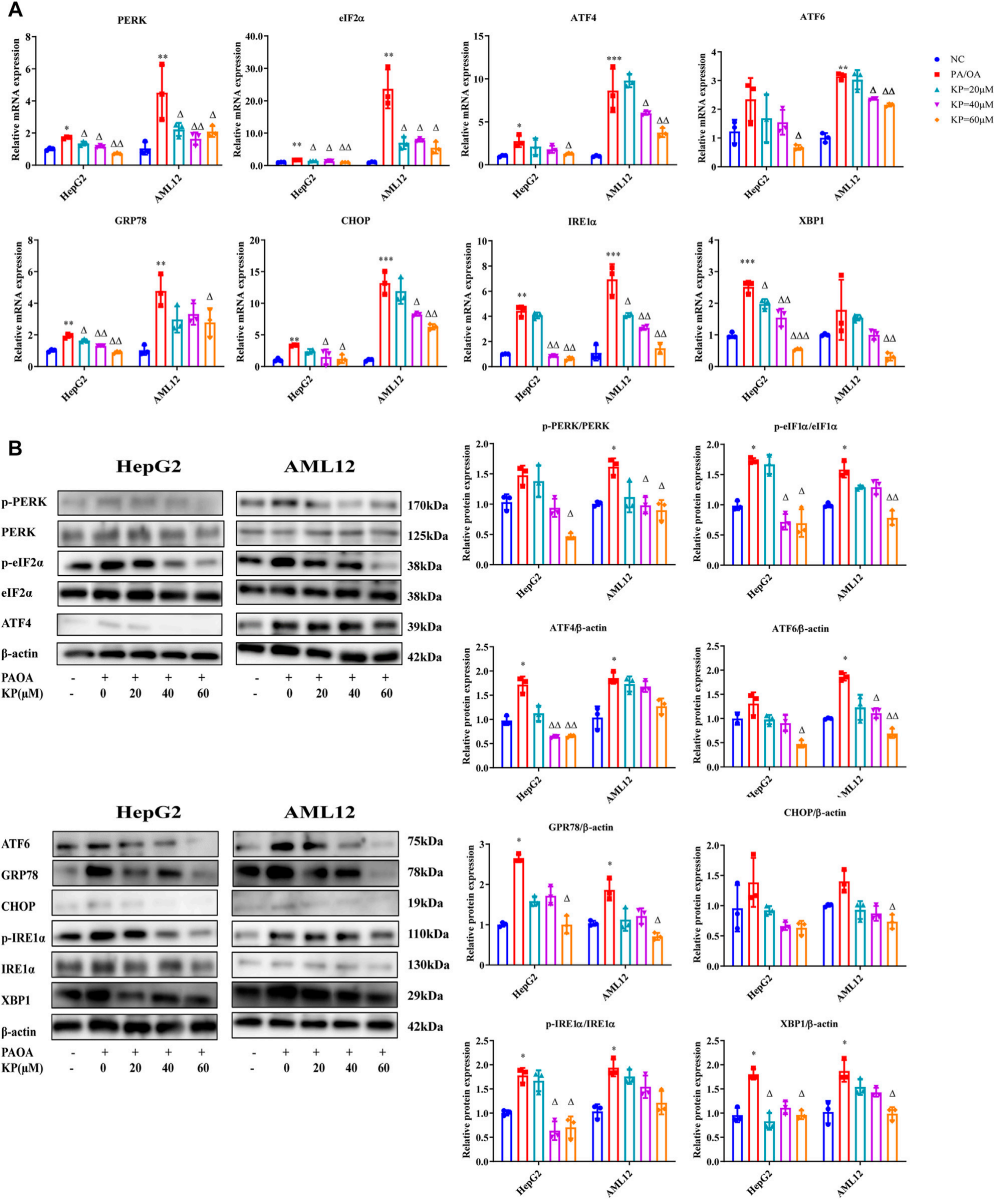
KP regulated the mRNA and protein expression of factors related to ERS *in vitro*. **(A)**: mRNA Expression in HepG2 and AML12 cells; **(B)**: protein Expression in HepG2 and AML12 cells. NC: Normal control group, PA/OA: PA/OA group, KP = 20 µM: KP low concentration group, KP = 40 µM: KP medium concentration group, KP = 60 µM: KP high concentration group. Quantification of mRNA and protein level expression was normalized to β-actin levels, except protein phosphorylation level expression of PERK, eIF2α, IRE1α expression was normalized to their prototypes. Compared with group NC, *, *p <* 0.05, **, *p <* 0.01, ***, *p <* 0.001; Compared with group PA/OA, △, *p <* 0.05, △△, *p <* 0.01.

Compared with group NC, the mRNA levels of PERK, eIF2α, ATF4, CHOP, ATF6, GRP78, XBP1 and IRE1α in group PA/OA were increased. The expression of PERK, eIF2α, ATF4, CHOP, ATF6, GRP78, XBP1 and IRE1α mRNA in the KP group were significantly reduced. After KP intervention, the down-regulation of eIF2α, ATF4, XBP1 and IRE1α mRNA in the two types of cells all showed concentration-dependent tolerance, whilst the down-regulation of PERK and CHOP mRNA was only concentration-dependent in HepG2 cells (Figure 6A).

The phosphorylation levels of PERK, eIF2α and IRE1α in the PA/OA group were higher than those in the NC group not only in HepG2 but also in AML12 cells, however the adjustments in the phosphorylation level of PERK in HepG2 cells were not statistically significant. The protein expressions of ATF4, CHOP, ATF6, GRP78 and XBP1 in the PA/OA group of the two cells also increased significantly. After the intervention of different concentrations of KP, the protein expression levels of these factors in the two cells were reduced, and the phosphorylation levels of PERK and eIF2α in the high-concentration group of KP decreased more significantly. In the two kinds of cells, the protein levels of ATF6, ATF4, CHOP, GRP78 and XBP1 were significantly reduced in the high concentration group. The phosphorylation level of IRE1α in the KP medium and high concentration groups of HepG2 cells extensively decreased, and the phosphorylation level of IRE1α in the KP intervention groups in AML12 cells showed a downward trend, but it was not obvious (Figure 6B).

### Kaempferol Reduced the mRNA Expression of Inflammatory Factors in Palmitic Acid/Oleic Acid-Induced Steatosis Model Cells

We also examined the mRNA expression of inflammation-related factors in cells using qRT-PCR.

**FIGURE 7:**
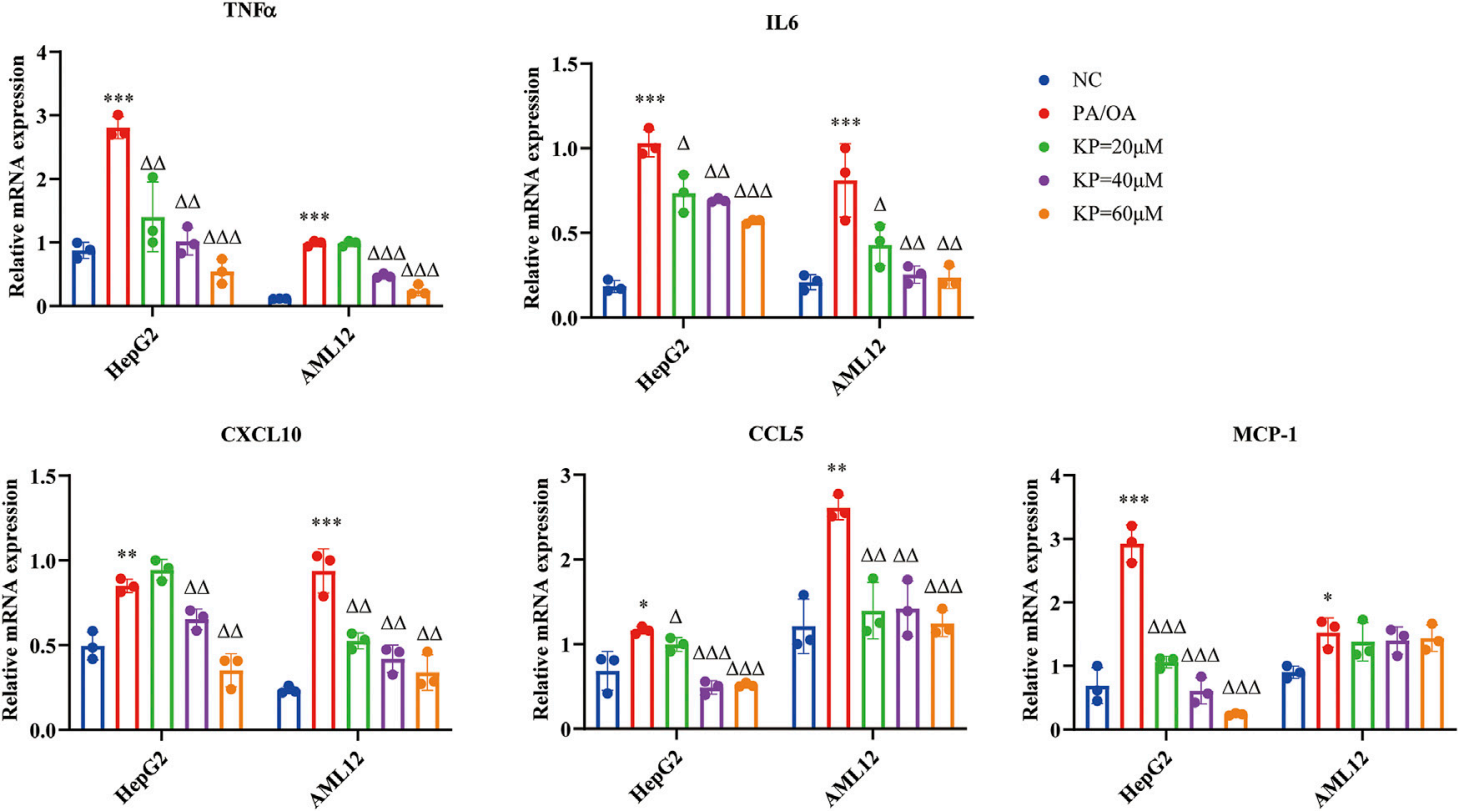
KP reduced the mRNA expression of inflammatory factors *in vitro*. NC: Normal control group, PA/OA: PA/OA group, KP = 20 µM: KP low concentration group, KP = 40 µM: KP medium concentration group, KP = 60 µM: KP high concentration group. Quantification of mRNA expression was normalized to β-actin levels. Compared with group NC, *, *p <* 0.05, **, *p <* 0.01, ***, *p <* 0.001; Compared with group PA/OA, △, *p <* 0.05, △△, *p <* 0.01, △△△, *p <* 0.001.

Compared with the NC group, the mRNA levels of TNFα, IL6, CXCL10, CCL5 and MCP-1 in the PA/OA group increased significantly in the two types of cells. In hepG2 cells, TNF-α, IL6, CCL5 and MCP-1 all showed a decrease in KP concentration dependent tolerance, and CXCL10 only decreased in the middle and high concentration groups of KP. In AML12 cells, the mRNA levels of IL6, CXCL10 and CCL5 were significantly down-regulated after KP intervention. TNFα did not decrease significantly when KP = 20 μM, but mRNA levels decreased significantly at KP = 40 and 60 μM. The expression of MCP-1 showed no obvious downward trend after KP intervention (Figure 7).

### Kaempferol Regulated Lipid Metabolism Both *in vivo* and *in vitro*

Peroxisome proliferator-activated receptor-α (PPAR-α) belongs to the PPAR subfamily, which is a type of nuclear receptor highly expressed in the liver. PPARα will be activated to enhance the oxidation of fatty acids when fatty acids in the liver are elevated (Pawlak et al., 2015). It was found that when liver PPARα was specifically knocked out, liver steatosis would increase (Montagner et al., 2016a). ATP binding cassette transporter A1 (ABCA1) is a transport protein involved in high-density lipoprotein (HDL) biosynthesis and cellular cholesterol homeostasis. Phospholipids and free cholesterol can be transferred to apolipoprotein A1 by ABCA1, and then form new HDL particles (Phillips, 2018). Previous studies have shown that overexpression of ABCA1 can reduce liver lipid levels (Yang et al., 2010; Liu et al., 2014). In the present study, we found that the mRNA and protein expression of PPAR-α and ABCA1 decreased in the HFD-induced NASH model than the control group, and also in the PA/OA-induced steatosis cell model, while KP could increase their expression both *in vivo* and *in vitro* (Supplementary Figure S3). These results suggest that KP plays a role in regulating lipid metabolism.

## Discussion

NAFLD, as one of the common diseases affecting human life and health in the world, has attracted more and more attention to researchers (Fan et al., 2017; Younossi et al., 2018). It is expected that the incidence of NAFLD would increase to 314.58 million by 2030 in China. Compared with the number of cases in 2016 (246.33 million cases), the increase was 29.1% (Estes et al., 2018). NASH is a type of NAFLD, and approximately 20% of NASH patients will develop liver cirrhosis. Now, NASH has become the main cause of liver transplantation in American patients (Sheka et al., 2020).

Our former study has shown that KP was effective against improving liver injury in HFD-induced NASH mice model (Lu et al., 2020). In the present study, we further demonstrated that KP can modulate the mRNA or the protein expression of the molecules involved in LXRα-LPCAT3-ERS pathway in both HFD-induced mice model and PA/OA-induced steatosis cell model. To our best knowledge, it is the first time to illustrate the molecular mechanism of KP on improving NASH.

KP is a type of aglycone flavonoids existing in a variety of plants, which has antibacterial, anti-inflammatory, antioxidant, anti-tumor, heart protection, neuroprotective and anti-diabetes effects (Chen and Chen, 2013; Dabeek and Marra, 2019; Imran et al., 2019). In our previous studies, it has been proved that KP can alleviate liver injury in HFD-induced NASH mice model (Lu et al., 2020). In this study, in order to verify the efficacy of KP on NASH, HepG2 and AML12 cell lines were selected to establish an *in vitro* cell model of steatosis using PA/OA. As similiar as the results *in vivo*, the content of TG in the steatosis model group decreased after the intervention of KP (20, 40, and 60 μM), in particular in the high concentration group, suggesting that KP attenuates the TG storage of PA/OA-induced steatosis cell model. From the results of oil red O staining, KP can also substantially decrease lipid deposition in fatty degeneration cells. These data further verified that KP can improve PA/OA induced liver steatosis.

The LXRα-LPCAT3 pathway is an important regulator of PLs metabolism, metabolic stress response and inflammation. Till now, there are different points on the role of LPCAT3 in the lipogenesis. LXR activation promotes the entry of unsaturated fatty acids into PLs through LPCAT3, thereby reducing liver ERS and inflammation (Rong et al., 2013). Patrick et al. (Ferrara et al., 2021) found that mice with skeletal muscle-specific knockout of LPCAT3 (LPCAT3-MKO) exhibited greater muscle lyso-PC/PC, concomitant with improved skeletal muscle insulin sensitivity. Conversely, skeletal muscle-specific overexpression of LPCAT3 (LPCAT3-MKI) promoted glucose intolerance. Thibaut et al. (Bourgeois et al., 2020) found that deletion of LPCAT3 in myeloid cells worsened hepatic steatosis after a high-fat diet. Recent study has shown that LPCAT3 is a direct PPAR-δ target gene and has a novel function of PPAR-δ in regulation of phospholipid metabolism through LPCAT3 (Singh and Liu, 2017a).

In this study, after the intervention of KP, the mRNA levels and protein expression levels of LXR and LPCAT3 were decreased in the liver tissues of the NASH mice model. Our studies supported that LPCAT3 was highly expressed in NASH model by the involvement of lipogenesis. The above studies support that KP can improve lipid accumulation through LXR-LPCAT3.

Wang et al. (2013) showed that LPCAT3 regulated the formation of ERS and inflammation. ERS is considered to be one of the necessary mechanisms that purpose liver steatosis and inflammatory changes (Rong et al., 2013). After ERS stimulation, the PERK/elF2α, ATF6 and IRE1/XBP-1 signal pathways are activated, and the mRNA and protein expression of PERK, eIF2α, ATF4, ATF6, IRE1α and GRP78 are considerably increased, which induces CHOP expression and promotes cell apoptosis (Wang and Kaufman, 2016). ERS will dissociate PERK from GPR78/bid, and then the downstream elF2α will be phosphorylated (Donnelly et al., 2013). Phosphorylated elF2α reduced the general translation rate, but activated the downstream translation of ATF4 and CHOP (Malhi and Kaufman, 2011; Luhr et al., 2019). The expression of the anti-apoptotic gene BCL-2 can be inhibited by CHOP, thereby accelerating cell death (Marciniak et al., 2004). ATF6 will transfer to the Golgi apparatus when it is activated. It becomes ATF6 (n) after being cleaved in the Golgi apparatus and regulates ERS together with XBP1s (Walter et al., 2018). The misfolded protein phosphorylates IRE1α and induces conformational changes in the RNase domain (Ghosh et al., 2014). Next, XBP1 is cut into XBP1s, which initiates the UPR gene expression program in response to ERS (Yoshida et al., 2001; Huang et al., 2019).

**FIGURE 8:**
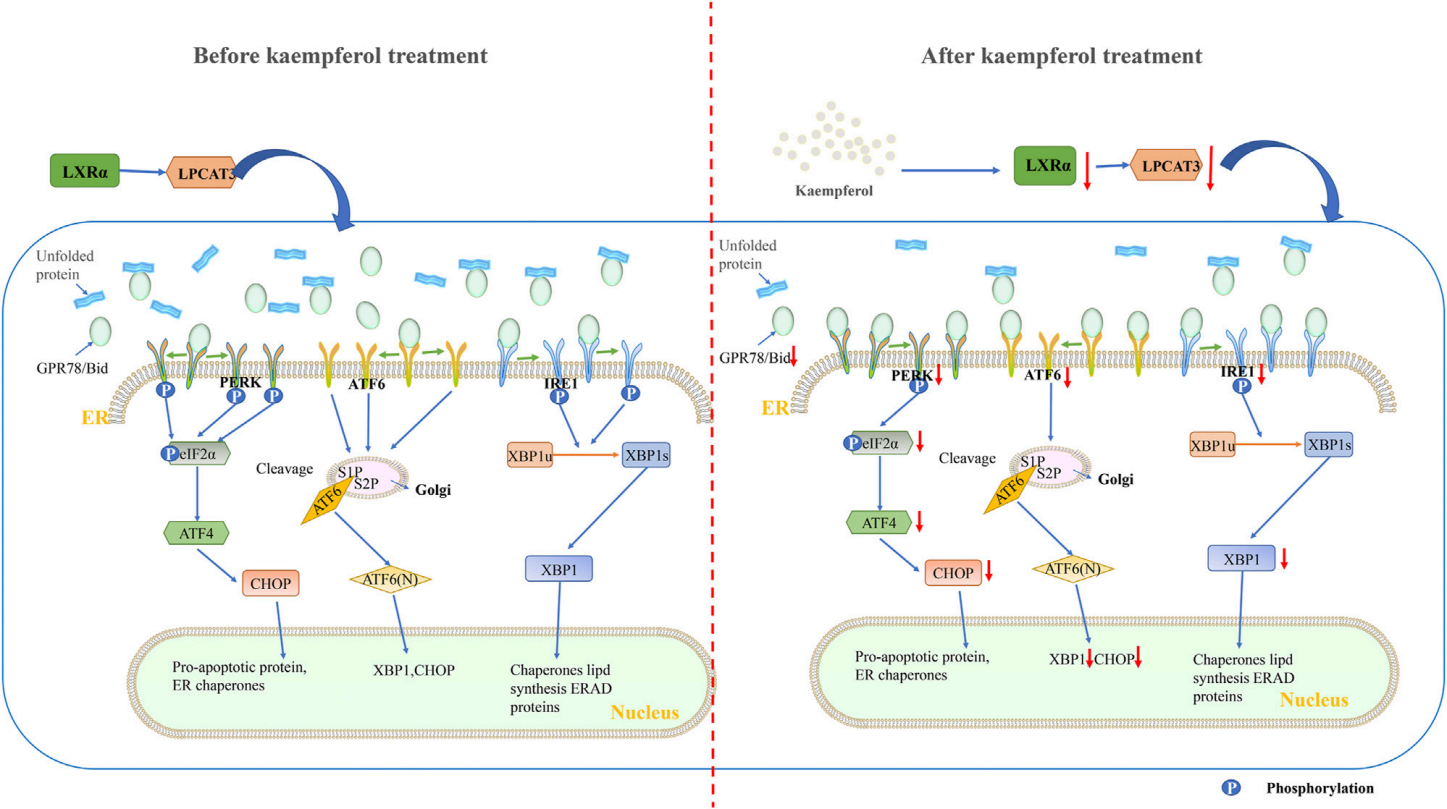
Schematic diagram of the mechanism of KP regulating LXRα-LPCAT3 signaling pathway. KP intervention suppressed endoplasmic reticulum stress. The LXR-LPCAT3-ERS pathway is activated when induced by HFD or PA/OA. This allows unfolded proteins to accumulate in the ER. At the same time, GPR78 dissociates from PERK, IRE1, and ATF6, and then binds to the unfolded protein. The dissociated PERK, IRE1 and ATF6 were activated. CHOP-induced apoptosis occurs when PERK, IRE1, ATF6 and their downstream pathways are continuously activated. The expression of LXR and LPCAT3 decreased significantly when treated with KP, and then the unfolded protein decreased, PERK, IRE1 and ATF6 dissociated from GRP78 decreased. Abbreviations: HFD: high fat diet, PA/OA: Palmitic acid/Oleic acid, LPCAT3: lysophosphatidylcholine acyltransferase 3, LXR α: liver X receptor alpha, PERK: protein kinase R-like ER kinase, Eif-2α: eukaryotic translation initiation factor 2α, ATF6: activating transcription factor 6, IRE1: inositol-requiring enzyme 1, XBP1: X-box-binding protein 1, ATF4: activating transcription factor 4, CHOP: C/EBP homologous protein, ERAD: Endoplasmic reticulum-associated degradation.

In our study, KP was used to interfere with NASH mouse model and PA/OA-induced steatosis model cells. It was found that after KP intervention, the expression of factors related to ERS decreased significantly from both mice and cells. First, the levels of PERK, eIF2α, ATF4 and CHOP in the PERK pathway were significantly decreased. Second, the expression of ATF6 in the ATF6 pathway also decreased. Third, the expression of IRE1α and XBP1 in the IRE1α pathway was also significantly inhibited. In addition, the expression of GPR78 was also suppressed, possibly because the dissociation of GPR78 from PERK, ATF6 and IRE1α became less. We concluded that KP regulates ERS status by reducing the expression of LXR-LPACT3, thereby improving lipid degeneration (Figure 8).

Cytokines are the main mediators required for the comprehensive response to various stimuli in the immune and inflammatory process. It includes interleukin, tumor necrosis factor, chemokine family, etc. These factors are key regulators that initiate and drive inflammatory diseases (Borish and Steinke, 2003). TNFα and IL6 are up-regulated in many inflammatory diseases, such as rheumatoid arthritis (van Schouwenburg et al., 2013; Pandolfi et al., 2020), inflammatory lung diseases (Malaviya et al., 2017) and tumors (Balkwill, 2006; Jones and Jenkins, 2018). Malaviya et al. (2017) showed that TNFα antagonists showed a good effect on inflammatory lung disease. Nakagawa et al. (2014) found that inflammatory macrophages activated by ERS promote the release of inflammatory mediators such as TNFα and IL6. In the liver, CCL5 and CXCL10 can regulate the cytopathic and antiviral immune response of T cells and natural killer cells. MCP-1 can promote the accumulation of macrophages, steatosis and inflammation (Marra and Tacke, 2014). Fan et al. (2020) found that CXCL10 promoted the recruitment of natural killer cells to the liver in the NASH model induced by the methionine-and choline-deficient diet (MCD). CCL5 was significantly up-regulated in HFD-induced liver fibrosis model (Li B.-H. et al., 2017). Baeck et al. (2012) found that blocking MCP-1 can reduced macrophage infiltration and reduced the release of pro-inflammatory factors TNFα, interferon g and IL6. Our results showed that KP can regulate the release of inflammatory factors in the NASH mouse model and cell model by improving ERS, and ultimately reduce liver inflammation.

 However, there are still several limitations in the present study. For example, LPCAT3 gene knockout animal models or siRNA interference techniques have not been used to further confirm the role of LPCAT3 in the mechanism of KP on improving NASH. Primary cells have not been used to build cell models. In a bioinformatics analysis study on NAFLD, it is mentioned that the occurrence of NAFLD is also related to insulin signaling pathway, PPAR signaling pathway, p53 signaling pathway and mitogen-activated protein kinase (MAPK) signaling pathway (Liu et al., 2020). The study found that giving p53 knockout mice a high-fat diet reduced the occurrence of NAFLD. Moreover, knocking out P53 in the cell also reduced the accumulation of lipids in the cell (Zhang et al., 2020). Tomita et al. (2012) showed that the expression of p53 and p66Shc was up-regulated in the liver tissue of the NASH mouse model. Montagner et al. (2016a) found that lack of PPARα in hepatocytes leads to susceptibility to steatosis, which may be related to the damage of fatty acid catabolism. Inhibition of MAPK signaling pathway also showed the effect of reducing liver lipid deposition and liver fibrosis in rats (Shen et al., 2019). It has been discovered that KP intervention affects the expression of PPARα and ABCA1, but the specific mechanism is still unclear. The role of the above pathways in the occurrence of NAFLD and NASH, and whether KP also plays a therapeutic role through these pathways will be the direction of our further research.

## Conclusion

In summary, this study has confirmed that KP can improve liver damage by regulating LXRα-LPCAT3 and inhibiting ERS from both *in vivo* and *in vitro*. At the same time, the expression of inflammatory factors is reduced. The role of KP provides a new direction for the treatment of NASH. LPCAT3 can also be used as a potential target for NASH treatment.

## Data Availability

The raw data supporting the conclusions of this article will be made available by the authors, without undue reservation, to any qualified researcher.

## Abbreviations

ABCA1, ATP binding cassette transporter A1; ALT, Alanine aminotransferase; ATF6, Activating transcription factor 6; BCA, Bicinchoninic acid; CCL5, C-C chemokine ligand 5; CCK8, Cell counting kit-8; CHOP, C/EBP homologous protein; CXCL10, C-X-C motif chemokine 10; eIF-2α, Eukaryotic translation initiation factor 2; ER, Endoplasmic reticulum; ERS, Endoplasmic reticulum stress; GPR78, Glucose-regulated protein 78; HFD, High-fat diet; HDL, High-density lipoprotein; IL6, Interleukin 6; IRE1, Inositol-requiring enzyme 1; KP, Kaempferol; LDL, Low density lipoprotein; LPCAT3, Lysophosphatidylcholine acyltransferase 3; LXRs, Liver X receptors; MAPK, Mitogen-activated protein kinase; MCP-1, Monocyte chemoattractant protein-1; NAFL, Non-alcoholic fatty liver; NAFLD, Non-alcoholic fatty liver disease; NASH, Non-alcoholic steatohepatitis; PA/OA, Palmitic acid/oleic acid; PERK, Protein kinase R-like ER kinase; PLs, Phospholipids; PPAR, Peroxisome proliferator-activated receptor; ROS, Reactive oxygen species; SREBP, Sterol regulatory element bingding proteins; TC, Total cholesterol; TG, Triglyceride; TNFα, Tumor necrosis factor α; UPR, Unfolded protein response; XBP1, X-box-binding protein 1.
