# Supplementary material for: Kaempferol Alleviates Steatosis and Inflammation During Early Non-Alcoholic Steatohepatitis Associated With Liver X Receptor α-Lysophosphatidylcholine Acyltransferase 3 Signaling Pathway
Source: Front Pharmacol. 2021 Jun 28;12:690736. doi: 10.3389/fphar.2021.690736 (PMC8273916; doi:10.3389/fphar.2021.690736)
Supplement: Supplementary file 1 [file DataSheet1.pdf]

Supplementary Material

Kaempferol alleviates steatosis and inflammation during early non-alcoholic steatohepatitis association with LXRα-LPCAT3 signaling pathway

Hongjiao Xiang[1,2#], Mingmei Shao[1,2#], Yifei Lu[1,2], Junmin Wang[1,2], Tao Wu[1,2*], Guang Ji[2*]

[1] Institute of Interdisciplinary Integrative Medicine Research, Shanghai University of Traditional Chinese Medicine, Shanghai, China; [2] Institute of Digestive Disease, Longhua Hospital, Shanghai University of Traditional Chinese Medicine, Shanghai, China
# These authors have contributed equally to this work.

*Correspondence to: Prof. Guang Ji, PhD. Institute of Digestive Disease, Longhua Hospital, Shanghai University of Traditional Chinese Medicine, South Wanping Road 725, Shanghai 200032, China. Email: jiliver@vip.sina.com; Prof. Tao Wu, PhD. Institute of Interdisciplinary Integrative Medicine Research, Shanghai University of Traditional Chinese Medicine, Cailun Road 1200, Shanghai 201203, China. Email: wutao001827@163.com.

List

Supplementary Figure 1. Scheme figure with experimental approach of the establishment of high-fat diet induced NASH C57BL/6J mouse model.
Supplementary Figure 2. The effect of different concentrations of PA/OA intervention on the cell viability, TG content and lipid droplet formation of HepG2 and AML12 cells.
Supplementary Figure 3. KP regulated lipid metabolism disorders both in vivo and in vitro.
Supplementary Table 1. Primers for real-time qPCR.

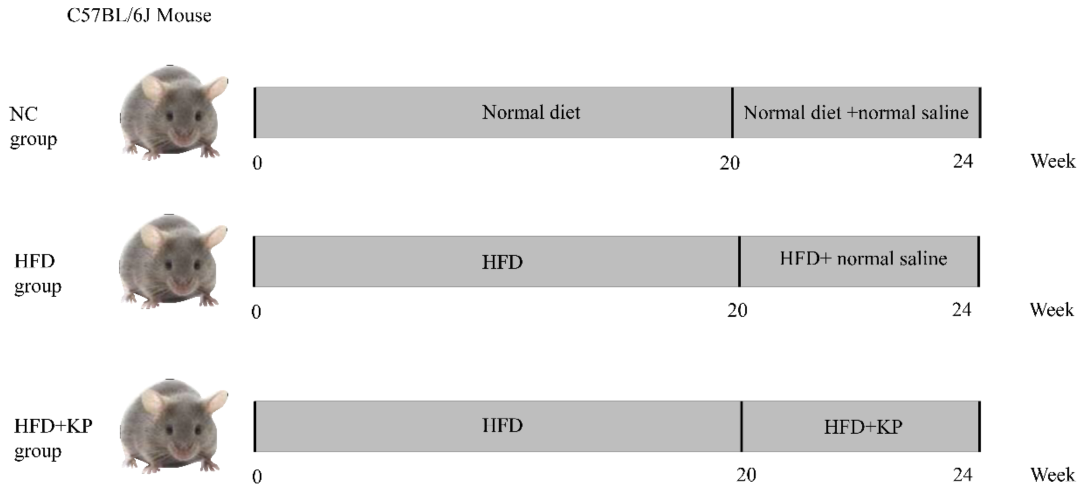

Supplementary Figure 1. Scheme figure with experimental approach of the establishment of high-fat diet induced NASH C57BL/6J mouse model. The mice in group NC were fed normal diet, and the mice in group HFD and HFD+KP were fed HFD for 24 weeks. At the beginning of the 21st week, KP (4mg/ml) was gavaged in the HFD + KP group at a dose of 0.5ml/100g on the basis of HFD feeding. At the same time, mice in NC and HFD groups received equal volume of normal saline.

NASH: non-alcoholic steatohepatitis; NC: normal control; HFD: high fat diet; KP: kaempferol.

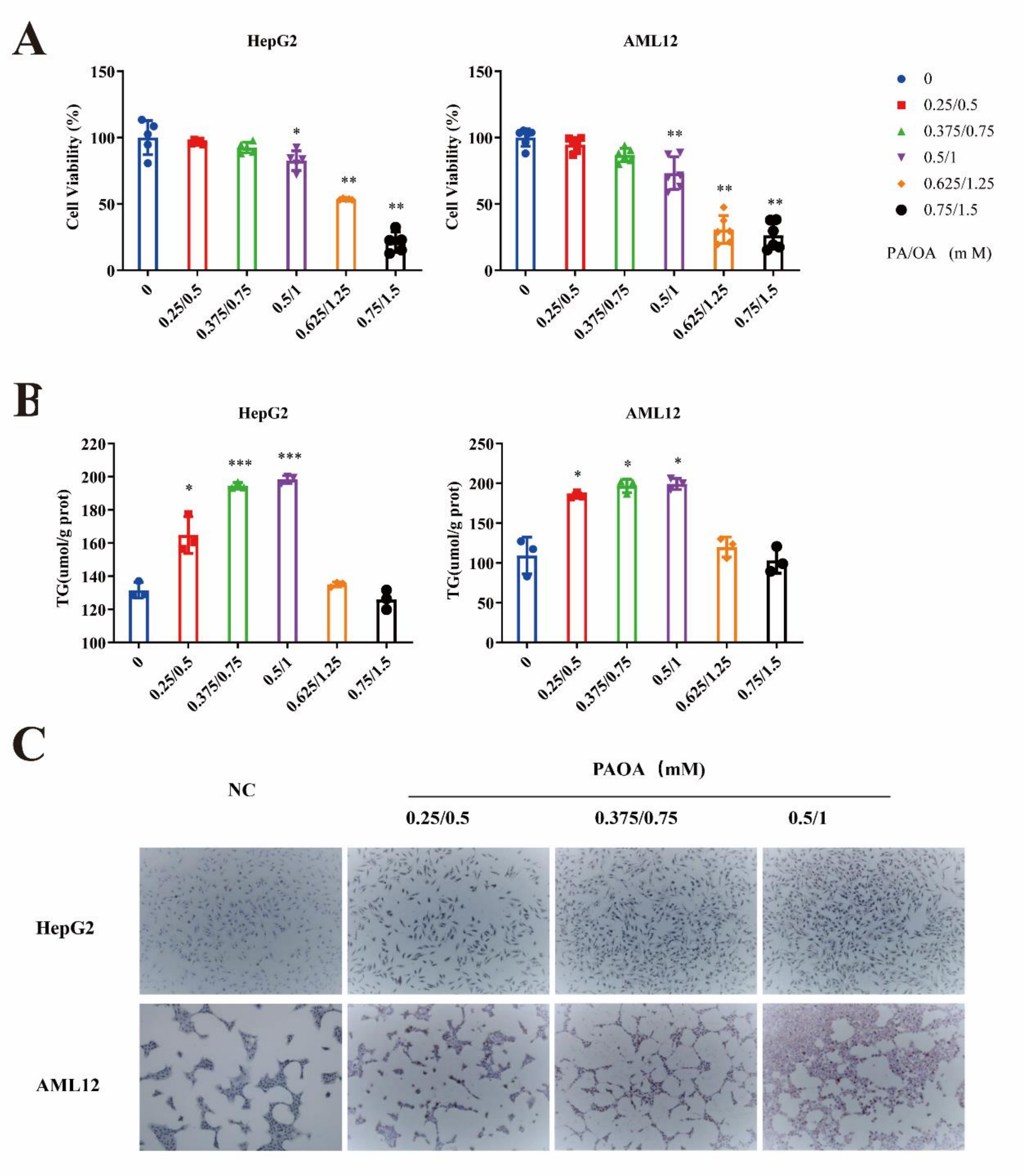

Supplementary Figure 2. The effect of different concentrations of PA/OA intervention on the cell viability, TG content and lipid droplet formation of HepG2 and AML12 cells. A: Changes in the survival rate; B: Changes of TG content; C: Oil red O staining in HepG2 and AML12 cells treated with different concentrations of PA/OA (x100).

NC: normal control group; PA/OA: PA/OA group.

Blue: PA/OA=0mM; Red: PA/OA=0.25/0.5mM; Green: PA/OA=0.375/0.75mM; Purple: PA/OA=0.5/1mM; Orange: PA/OA=0.625/1.25mM; Black: PA/OA=0.75/1.5mM.

Compared with group PA/OA=0mM, *, P<0.05, **, P<0.01, ***, P<0.001.

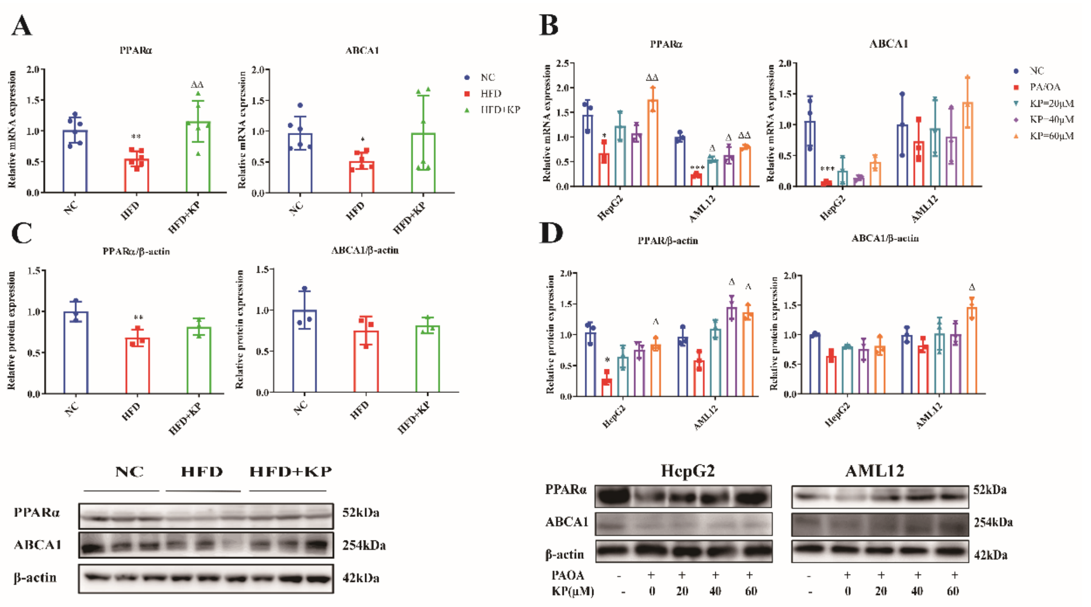

Supplementary Figure 3. KP regulated lipid metabolism disorders both in vivo and in vitro. A: PPARα and ABCA1 mRNA levels in vivo; B: PPARα and ABCA1 mRNA levels in vitro; C: The protein expression of PPARα and ABCA1 in vivo; D: The protein expression of PPARα and ABCA1 in vitro. Quantification of mRNA and protein level expression was normalized to β-actin levels. PPARα : peroxisome proliferator-activated receptor α; ABCA1 : ATP binding cassette transporter A1.

NC: normal control group; HFD: HFD group; HFD+KP: HFD+KP group; PA/OA: PA/OA group; KP: KP group; KP=20μM: KP low concentration group; KP=40μM: KP medium concentration group; KP=60μM: KP high concentration group. Compared with group NC, *, P<0.05, **, P<0.01. In Supplementary Figures 3A and C, compared with group HFD, △, P<0.05, △△, P<0.01. In Supplementary Figures 3B and D, compared with group PA/OA, △, P<0.05, △△, P<0.01.

Supplementary Table 1. Primers for real-time qPCR.

Genes	Sequence (5'to 3')	Base pair (bp)
mActin	mForwardPrimer 5'-GAGACCTTCAACACCCCAGC-3'	263
	mReverse Primer 5'-ATGTCACGCACGATTTCCC-3'	
mLXRα	mForwardPrimer 5'-CCAAAATGCTGGGGAACG-3'	125
	mReverse Primer 5'-GCGTGCTCCCTTGATGACA-3'	
mLPCAT3	mForwardPrimer 5'-CCCCACATCACAGACGACTATC-3'	192
	mReverse Primer 5'-TCTCACGGTCCCATTTTCATC-3'	
mPERK	mForwardPrimer 5'-GCTCAAAGACGAAAGCACAGAC-3'	153
	mReverse Primer 5'-CCCACCGAGAAAGACCGAC-3'	
meIF2α	mForwardPrimer 5'-ACCTGGATACGGTGCCTACG-3'	145
	mReverse Primer 5'-TCGAATTTTGACCGCTTGTG-3'	
mATF4	mForwardPrimer 5'-ATGGAGCAAAACAAGACAGCA-3'	180
	mReverse Primer 5'-TGCCTTACGGACCTCTTCTATC-3'	
mCHOP	mForwardPrimer 5'-AAACCTTCACTACTCTTGACCCTG-3'	184
	mReverse Primer 5'-GGGCACTGACCACTCTGTTTC-3'	
mATF6	mForwardPrimer 5'-AAAGTCCCAAGTCCAAAGCG-3'	116
	mReverse Primer 5'-CTAGGTTTCACTCTTCGGGATTC-3'	
mGRP78	mForwardPrimer 5'-TTGTCCCCTTACACTTGGTATTG-3'	170
	mReverse Primer 5'-TGTCTTTTGTTAGGGGTCGTTC-3'	
mIRE1α	mForwardPrimer 5'-TTTGTTTTCCGAGGACAGTTTG-3'	136
	mReverse Primer 5'-AGAAGTAGCGAAGCACGTTGG-3'	
mXBP1	mForwardPrimer 5'-CAAGTGGTGGATTTGGAAGAAG-3'	119
	mReverse Primer 5'-TCCATTCCCAAGCGTGTTC-3'	
mPPARα	mForwardPrimer 5'-CCTGAAAGATTCGGAAACTGC-3'	139
	mReverse Primer 5'-GACAAAAGGCGGGTTGTTG-3'	
mABCA1	mForwardPrimer 5'-CCTGTGGGGGCATCATCTAC-3'	178
	mReverse Primer 5'-TCCCATTGGACCCCGATAC-3'	
mMCP-1	mForwardPrimer 5'-GCTGACCCCAAGAAGGAATG-3'	184
	mReverse Primer 5'-TTGAGGTGGTTGTGGAAAAGG-3'	
mCCL5	mForwardPrimer 5'-ACCACTCCCTGCTGCTTTG-3'	129
	mReverse Primer 5'-CACTTGGCGGTTCCTTCG-3'	
mCXCL10	mForwardPrimer 5'- AAGTGCTGCCGTCATTTTCTG-3'	160
	mReverse Primer 5'- GGATAGGCTCGCAGGGATG-3'	
mTNF-α	mForwardPrimer 5'- CCCTCCAGAAAAGACACCATG-3'	183
	mReverse Primer 5'- CACCCCGAAGTTCAGTAGACAG-3'	
mIL6	mForwardPrimer 5'- AAATGATGGATGCTACCAAACTG-3'	137
	mReverse Primer 5'- CTCTGGCTTTGTCTTTCTTGTTATC-3'	
hActin	hForwardPrimer 5'- CTCCATCCTGGCCTCGCTGT-3'	268
	hReverse Primer 5'- GCTGTCACCTTCACCGTTCC-3'	
hLXRα	hForwardPrimer 5'-GAAACTGAAGCGGCAAGAGG-3'	152
	hReverse Primer 5'-AGCGCCGGTTACACTGTTG-3'	

hLPCAT3	hForwardPrimer 5’-GAAAAGGGCAAGGCAAAGTG-3’	114
	hReverse Primer 5’- CCAGGCGTTGGTGTTGATG-3’	
hPERK	hForwardPrimer 5’-CTCGGGAAAAGGTAATGCG-3’	119
	hReverse Primer 5’-ATCCATCTTTTCTTGCCACTTC-3’	
heIF2α	hForwardPrimer 5’-GATTGAGGAAAAGAGGGGTGTG-3’	154
	hReverse Primer 5’- TTTGGCTTCCATTTCTTCTGC-3’	
hATF4	hForwardPrimer 5’-CTCAGCACAGCCCCTCTACC-3’	141
	hReverse Primer 5’- CCAGTTTCTCACCCTTTACTTTTG-3’	
hCHOP	hForwardPrimer 5’-AACCAGGAAACGGAAACAGAG-3’	192
	hReverse Primer 5’- TTCACCATTCGGTCAATCAGA-3’	
hATF6	hForwardPrimer 5’-CTCAGCACAGCCCCTCTACC-3’	125
	hReverse Primer 5’-CCAGTTTCTCACCCTTTACTTTTG-3’	
hGRP78	hForwardPrimer 5’-GTCCTATGTCGCCTTCACTCC-3’	137
	hReverse Primer 5’-GCACAGACGGGTCATTCCAC-3’	
hIRE1α	hForwardPrimer 5’-GGAATTACTGGCTTCTGATAGGAC-3’	177
	hReverse Primer 5’-GTGCGTTTTCTGAAGTCTGGTC-3’	
hXBP1	hForwardPrimer 5’-ATGGATTCTGGCGGTATTGAC-3’	175
	hReverse Primer 5’-GAGAAAGGGAGGCTGGTAAGG-3’	
hPPARα	hForwardPrimer 5’- GCGAACGATTCGACTCAAGC-3’	119
	hReverse Primer 5’-ACATCCCGACAGAAAGGCAC-3’	
hABCA1	hForwardPrimer 5’-CTGCTAATTGCCAGACGGAG-3’	143
	hReverse Primer 5’-TACATCCAGGGCTGAAGTTCC-3’	
hMCP-1	hForwardPrimer 5’- CCTTCTGTGCCTGCTGCTC -3’	171
	hReverse Primer 5’- GCTTCTTTGGGACACTTGCTG -3’	
hCCL5	hForwardPrimer 5’- ACCACACCCTGCTGCTTTG -3’	168
	hReverse Primer 5’- GATGTACTCCCGAACCCATTTC -3’	
hCXCL10	hForwardPrimer 5’- TATTCCTGCAAGCCAATTTTG -3’	129
	hReverse Primer 5’- CTTTCCTTGCTAACTGCTTTCAG -3’	
hTNF-α	hForwardPrimer 5’- CACGCTCTTCTGCCTGCTG -3’	129
	hReverse Primer 5’- GGCTTGTCACTCGGGGTTC -3’	
mIL6	hForwardPrimer 5’- AAAGCAGCAAAGAGGCACTG -3’	137
	hReverse Primer 5’-TACCTCAAACTCCAAAAGACCAG -3’	
